# Synchronous cardiac arrest in monozygotic twins with hypertrophic cardiomyopathy - Is sudden cardiac death genetically pre-programmed?

**DOI:** 10.1186/s12872-015-0007-3

**Published:** 2015-02-28

**Authors:** Cheng Yee Goh, Muhammad Asrar ul Haq, Vivek Mutha, William J van Gaal

**Affiliations:** Department of Cardiology, The Northern Hospital, Melbourne, Victoria Australia; The University of Melbourne, Melbourne, Victoria Australia

**Keywords:** Hypertrophic cardiomyopathy, Cardiac arrhythmia, Implantable cardioverter-defibrillator, Sudden cardiac death

## Abstract

**Background:**

Hypertrophic cardiomyopathy (HCM) is a myocardial disorder characterised by left ventricular hypertrophy (LVH) in the absence of another cardiac or systemic disease capable of producing the magnitude of LVH evident. HCM causes variable symptoms and is one of the leading causes of sudden cardiac death (SCD) in young adults. While various phenotypic features of HCM among monozygotic twin pairs are not uncommonly reported, occurrence of synchronous cardiac arrest among them is not known from literature.

**Case presentation:**

We present a case of monozygotic twins with HCM who both had a cardiac arrest post physical exertion in 63^rd^ year of their lives.

**Conclusion:**

This case highlights potential genetics predisposition of cardiac arrest in patients with HCM despite having different phenotypic expression. SCD may be the only manifestation of patients with HCM. Decision of implantable cardioverter-defibrillator (ICD) placement for primary prevention of SCD should be based on the recommended guidelines, clinical judgment and patient’s preference.

## Background

HCM is a myocardial disorder characterised by left ventricular hypertrophy (LVH) in the absence of another cardiac or systemic disease capable of producing the magnitude of LVH evident. It is a genetic disorder, most commonly caused by autosomal dominant mutations in genes encoding protein components of sarcomere and myofilaments [1], characterised by variable disease expression and age-related penetrance [2]. HCM causes variable symptoms and is one of the leading causes of SCD in young adults [3]. We present a case of monozygotic twins with HCM who both had a cardiac arrest post physical exertion in 63^rd^ year of their lives.

## Case presentation

A 62-year-old man presented with first episode of unconscious collapse secondary to malignant ventricular tachyarrhythmia with underlying HCM. Fortunately he survived the episode as he had an implantable cardioverter-defibrillator (ICD) inserted two months ago, after his monozygotic twin brother suffered a fatal cardiac arrest.

Patient’s twin brother, in whom the basis of monozygosity was established by history taken from our patient, was diagnosed with HCM 30 years ago following a routine work-related medical check-up. He was a physically active person, with no known risk factors for ischaemic heart disease (IHD) and no regular medication. He had an excellent exercise tolerance including regular strenuous activities like surfing and skiing. He did not have any prior history of syncope or presyncope, or any family history of sudden death. His echocardiogram done 4 weeks prior to event showed normal left ventricular (LV) size and function, marked asymmetric septal hypertrophy of 1.9 cm and no evidence of left ventricular outflow tract (LVOT) obstruction. A 24-hour holter monitoring done 4 years ago showed no evidence of non-sustained ventricular tachycardia (NSVT). The only available stress echocardiogram done 9 years ago showed no inducible LVOT gradient with a normal blood pressure response. He suffered sudden cardiac death at the age of 62 when he collapsed immediately after completing few laps of swimming with pre-warning signs of dizziness.

**Figure 1:**
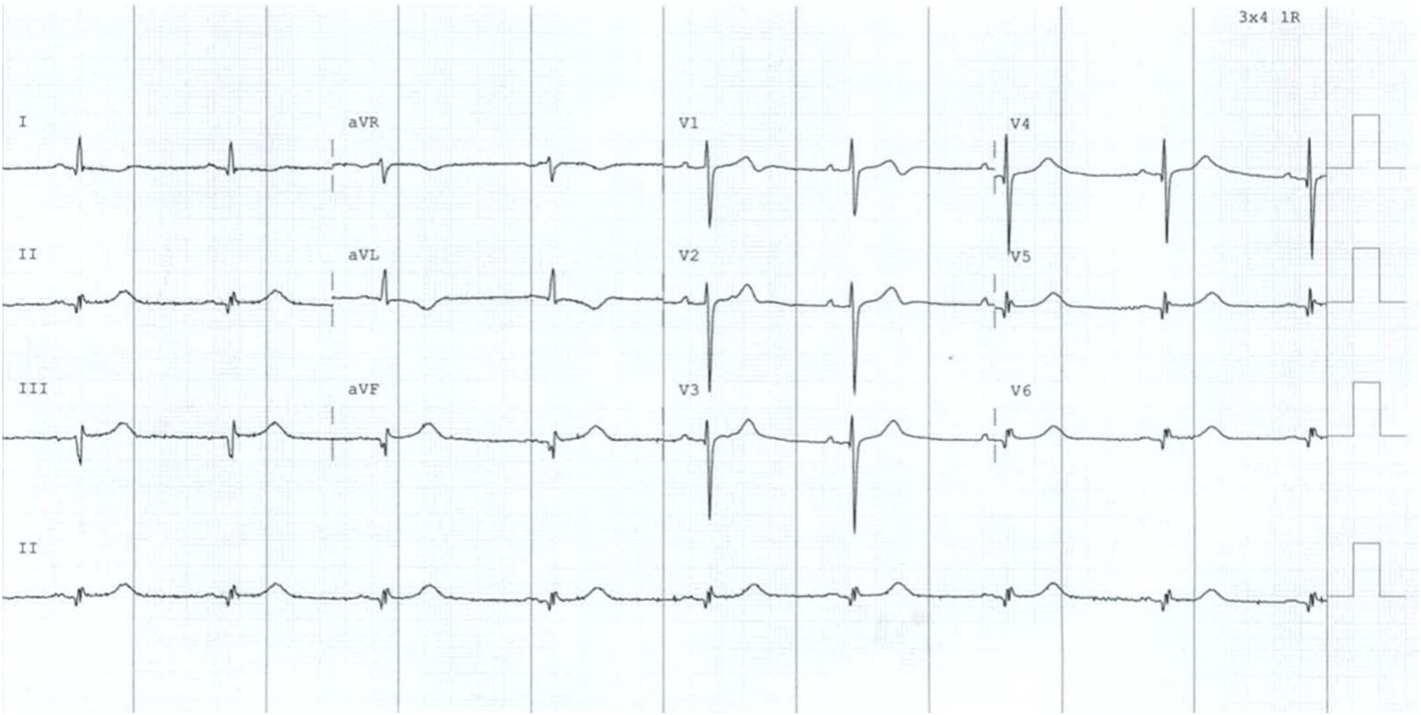
**Patient’s 12-lead ECG on arrival to hospital showing sinus rhythm.**

**Figure 2:**
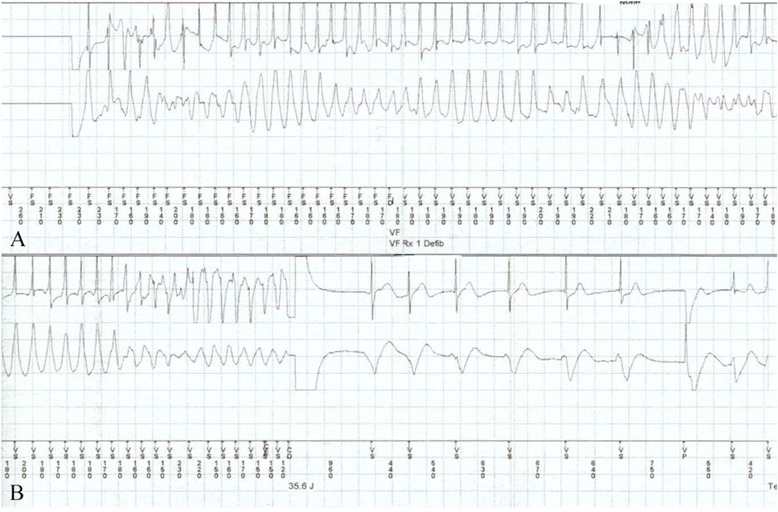
**Interrogation of implantable cardioverter-defibrillator (ICD). A**. Rhythm strip showing ventricular tachyarrhythmia. **B**. Ventricular tachyarrhythmia successfully terminated by the ICD shock.

Our patient was also known to have asymptomatic HCM as part of family screening 30 years ago. Following his brother’s death, he underwent an elective ICD implantation a week later. After two months, he presented to the hospital with an unwitnessed syncopal episode lasting one minute, preceded by pre-warning signs of dizziness while at work on a ladder. He regained his consciousness afterwards and rang an ambulance. The ECG revealed a sinus rhythm with normal axis (Figure 1). Interrogation of ICD revealed 15 episodes of NSVT with a rate of 140 beats/min, followed by malignant ventricular tachyarrhythmia for 12 seconds, terminated successfully by a single shock (Figure 2).

Like his brother he was previously well with no significant cardiovascular risk factors for IHD and no regular medication. His physical examination was unremarkable with vital parameters within normal limits. His initial troponin was 0.06 (NR <0.04) with no serial increment. An urgent angiogram revealed no significant coronary artery disease. An echocardiogram two months before the incident showed normal LV size and systolic function, asymmetric septal hypertrophy of 2.8 cm, and no evidence of LVOT obstruction. His previous 24-hour holter monitoring done three years ago had shown no evidence of NSVT. He did not undergo any exercise test in the past 8 years.

He was admitted to intensive care unit (ICU) with cardiac monitoring and was commenced on sotalol. There was no further ventricular tachyarrhythmias noted on telemetry and he was discharged on day 4 with outpatient follow up.

### Differential diagnosis

As patient’s brother did not undergo a post-mortem examination, the exact cause of death could not be ascertained. Differential diagnosis to consider apart from sudden cardiac death secondary to HCM would include coronary artery disease or pulmonary embolism. For our patient, ventricular tachyarrhythmia arising from a proarrhythmic impulse from the recently implanted ICD could be an alternative explanation for his ventricular arrhythmia episode.

### Discussion

The exact mechanism of SCD in HCM is not known, but is thought to be secondary to malignant ventricular tachyarrhymias arising from abnormal myocardial fibres [4]. While various phenotypic features of HCM among monozygotic twin pairs are not uncommonly reported [5], occurrence of synchronous cardiac arrest among them is not known from literature.

As SCD may be the only manifestation of patients with HCM, it is important to distinguish features of HCM, which are associated with high risk of SCD. The consensus document of American College of Cardiology Foundation and American Heart Association classified these risk factors as established risk markers and potential SCD risk modifiers [6]. Established risk markers include previous cardiac arrest (ventricular fibrillation; VF) or sustained VT, family history of SCD at age < 50, unexplained syncope, abnormal exercise blood pressure, and LV thickness greater than 30 mm (Table 1). The potential risk modifiers include presence of LVOT obstruction, LV apical aneurysm, late gadolinium enhancement (LGE) in cardiac magnetic resonance imaging (MRI) and high risk mutations. Furthermore, the guidelines recommend risk stratification be performed periodically every 12 to 24 months for patients with HCM who have not undergone an ICD implantation (Class IIa). Invasive electrophysiological study is not recommended in accessing SCD risk in HCM [6].

**Table 1:** **Indications for ICD implantation in HCM patients to prevent SCD**

**ICD Recommended (Class I)**	**ICD Reasonable (Class IIa)**	**ICD May be Useful (Class IIa)**
● Prior cardiac arrest	● Family history of sudden death in first degree relative (age <50)	● Non-sustained VT or abnormal BP response in presence of other SCD risk modifiers.
● Sustained VT	● LV wall thickness ≥ 30 mm	Note: Role of ICD is uncertain in non-sustained VT or abnormal BP response in absence of other SCD risk modifiers (Class IIb)
	● Recent unexplained syncope	

Modified from *Gersh BJ, Maron BJ, Bonow RO et al. 2011 ACCF/AHA Guideline for the Diagnosis and Treatment of Hypertrophic Cardiomyopathy: A Report of the American College of Cardiology Foundation/American Heart Association Task Force on Practice Guidelines. Circulation. 2011;* ***124*** *: e783-e831*.

(ICD = implantable cardioverter-defibrillator; HCM = hypertrophic cardiomyopathy; SCD = sudden cardiac death; VT = ventricular tachycardia; LV = left ventricle/ventricular; BP = blood pressure).

Though currently available genetic testing is considered reasonable to identify first-degree relative at risk of developing HCM [6], it has not been proven useful in predicting clinical course and risk of SCD [7]. Family members with similar genetic mutations may have entirely different phenotypic expression [8]. While most phenotype of HCM in monozygotic twins are concordant, phenotype with discordant features has been reported [9]. Our case report relates to twin brothers who had varying severity of asymmetrical LV hypertrophy, lived different life styles in two different geographical areas of Australia, and suffered cardiac arrest around the same time in their lives. This highlights the potential genetic predisposition of cardiac arrest in patients with HCM despite having different phenotypic expression. While it is likely to be a coincidence, the synchronous timing of cardiac arrest does raise a question whether an alternative explanation such as an underlying genetic cause could account for such synchronous timing. This however remains unlikely as lacks scientific evidence at this stage, and if such genetic factor did exist, more cases of synchronous cardiac arrests among siblings or relatives would have been reported in literature.

## Conclusion

This case highlights potential genetic predisposition of cardiac arrest in patients with HCM despite having different phenotypic expression. SCD may be the only manifestation of patients with HCM. Decision of ICD placement for primary prevention of SCD should be based on the recommended guidelines, clinical judgment and patient’s preference.

## Consent

Written informed consent was obtained from our patient and also from the deceased’s wife for publication of this case report and any accompanying images. Copies of the written consents are available for review by the Editor of this journal.
